# Preparation of a Periodic Polystyrene Nanosphere Array Using the Dip-Drop Method with Post-deposition Etching and Its Application of Improving Light Extraction Efficiency of InGaN/GaN LEDs

**DOI:** 10.1186/s11671-018-2595-1

**Published:** 2018-06-15

**Authors:** Po-Hsun Lei, Chyi-Da Yang, Yong-Sian Yang, Jian-Hong Lin

**Affiliations:** 1 Institute of Electro-Optical and Materials Science, National Formosa University, 64 Wen-Hwa Rd, Hu-Wei, 623 Yun-Lin Taiwan; 2 Department of Microelectronic Engineering, National Kaohsiung Marine University, Kaohsiung, 811 Taiwan

**Keywords:** Polystyrene nanospheres, Dip-drop method, Post-deposition etching, InGaN/GaN LEDs

## Abstract

In this study, we synthesized a periodic polystyrene nanosphere (PS NS) array using the dip-drop method with post-deposition etching to improve the light extraction efficiency (LEE) of InGaN/GaN light-emitting diodes (LEDs). The dip-drop method has advantages such as simple procedure, inexpensive equipment, room temperature deposition, and easy implementation in LEDs. The arrangement of PS NSs on an indium-tin-oxide (ITO)-coated glass substrate depends on the average dip-drop speed and the concentration of the PS NS suspension. The periodic PS NS array can modulate the in-plane wave vector of emission light from a semiconductor to free space and thus increase the escape probability. The calculated and experimental results indicated that the light output intensity of the InGaN/GaN LEDs can be improved by using the periodic PS NS array as a window layer; this array comprises PS NSs with a diameter of 100 nm separated with periods of 100 and 100 nm in the *x* and *y* directions. Because of the improved LEE, the InGaN/GaN LEDs with the optimal PS NS array window layers exhibited a 38% increase in light output intensity compared with the conventional InGaN/GaN LEDs under 20-mA driving current.

## Background

Recently, photonic crystals (PCs) have been widely investigated to improve the efficiency of optoelectronic devices such as light-emitting diodes (LEDs) [1], solar cells [2], and photodetectors [3]. PCs are structures in which a periodic variation in the refractive index occurs at the scale of the wavelength of light in one or more directions [4, 5]. The structure of PCs with a sufficiently large refractive index contrast can yield a photonic bandgap in which the frequency range of propagating light is forbidden. The light extraction efficiency (LEE) of LEDs can be improved by using the PCs through two methods. One approach is to design the PC structure with a bandgap to match the trapped waveguide modes within the LED. The waveguide light within the bandgap of the PC is blocked in the lateral direction in the structure and guided to the only external emission channel for the light to exit the device. However, this approach is difficult to realize because of the significant material processing problem of creating a planar structure with a sufficiently large refractive index contrast to open a full optical bandgap. Another approach is to utilize the periodic refractive index of the PC to diffract the waveguide mode above a certain cutoff frequency into externally propagating modes: **k**_**‖m**_ = **k**_**‖**_ + n**k**_**pc**_, where **k**_**‖m**_ and **k**_**‖**_ are the modified and original in-plane wave vectors, respectively; n is an integer; and **k**_**pc**_ is the reciprocal wave vector depending on the PC lattice constant. When the periodicity is chosen correctly, the modified in-plane wave vector falls within the escape corn, resulting in extraction to air at an angle dependent on the specific lattice constant within this range. Several methods exist to define the periodic PC structures on indium-tin-oxide (ITO) or p-GaN, including electron beam lithography [6–9], laser holographic lithography [10], focused ion beam technology [11], nanoimprint lithography [12], and self-assembled colloidal polystyrene nanosphere (PS NS) coating [13, 14]. The self-assembled PS NS coating method has advantages such as a large area arrangement with a gradually changing fill factor, simple process, sophisticated equipment, and etching damage.

Gallium nitride-based LEDs with wavelengths from ultraviolet to blue/green have attracted considerable research attention [15, 16]. GaN-based LEDs with high brightness can be used in applications such as large-size full-color displays, short-haul optical communication, traffic signal lights, and backlights for color liquid crystal displays [17–19]. The brightness of GaN-based LEDs depends on the external quantum efficiency (EQE), which is the product of internal quantum efficiency and LEE. Because of the inherently high refractive index contrast between free space and the semiconductor material, the calculated critical angle for the generated light to escape from the p-GaN layer into the air is approximately 23°. The small critical angle indicated that few photons can be extracted from the device due to the total internal reflection (TIR). Thus, the LEE of GaN-based LEDs is very low, leading to a low EQE for GaN-based LEDs. Several studies [20–23] have employed textured or patterned sapphire as a back reflector to increase the number of escape photons. The LEE for GaN-based LEDs with textured or patterned sapphire can be improved by the high probability of photons reflected from sapphire. However, the mechanically and chemically strong nature of sapphire renders roughening and patterning a challenging task. In addition, achieving the small dimensions of scattering objects through photolithography is difficult because of the short wavelength of nitride-based LEDs. Studies [24–26] have reported that a textured GaN surface can be used to increase the critical angle to enhance the LEE. However, surface texturing of GaN-based LEDs is impeded by the thin p-GaN and the sensitivity of p-GaN to plasma damage and electrical deterioration. In addition to the textured GaN surface, some studies [27, 28] have attempted to roughen the mesa sidewalls through photochemical etching or create oblique mesa sidewalls through a reflowed photoresist and adjust the CF_4_ flow during dry etching to increase the LEE. However, the surface of the rough mesa sidewalls was nonuniform, and the improved LEE for oblique mesa sidewalls was restricted within the sidewall region [29].

In this study, we investigated the conditions for compact and periodic PS NS array on an ITO surface using the dip-drop method with post-deposition etching and performed parametric analysis to optimize the LEE of InGaN/GaN LEDs with the periodic PS NS array. The deposition parameters of the compact PS NS array are the dip-drop speed and the concentration of the PS NS suspension. The calculated results indicate that the LEE of InGaN/GaN LED is related to the PS NS diameter and period of PS NSs. The InGaN/GaN LEDs with and without an optimal periodic PS NS array on ITO are compared.

## Experimental

### Dip-Drop Method

**Fig. 1:**
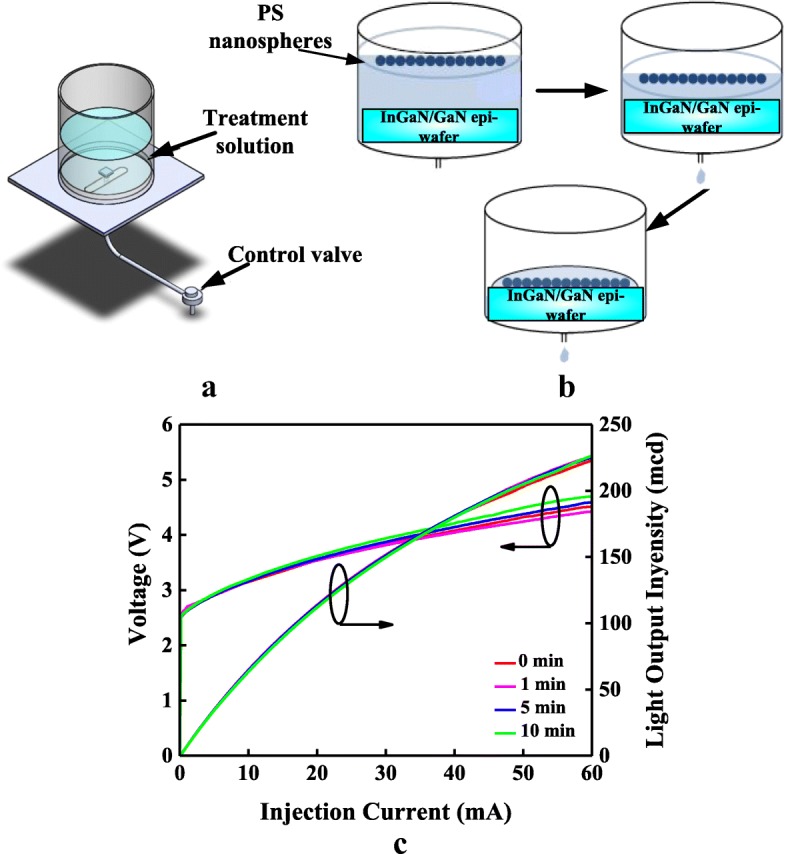
(**a**) Equipment, (**b**) process of the dip-drop method, and (**c**) I-V and L-I of the InGaN/GaN LEDs with different oxygen plasma-treated times

The equipment required to obtain a periodic PS NS array on InGaN/GaN LED through the dip-drop method is very simple and easy to prepare. It comprises a glass container with a hole at the bottom (main container) and a tuning control valve connected to the hole, as shown in Fig. 1 (**a**). Different volumes of deionized (DI) water and a PS NS colloidal suspension (Echo Chemical Co., USA) were mixed in the glass container, and this mixture was stirred for several minutes to obtain a PS NS suspension with a specific concentration. Three types of PS NS colloidal suspension including PS NSs with diameters of 100, 200, and 500 nm were diluted for the dip-drop process. After stirring, the PS NS suspension was added to the main container. The tuning control valve shown in Fig. 1 (a) was used to modulate the dip-drop speed of PS NS suspension. Figure 1 (b) shows the schematic dip-drop process for InGaN/GaN LEDs with a compact PS NS array window layer. First, an InGaN/GaN epi-wafer, which was treated with oxygen plasma to obtain a hydrophilic surface, was placed at the bottom of the main container, which contained the PS NS suspension at a specific concentration. Second, the PS NS suspension was filtered through the control valve at a constant dip-drop speed, and the PS NSs were then distributed on the surface of InGaN/GaN epi-wafer. Finally, the self-assembling PS NS array was formed on the InGaN/GaN epi-wafer after a room temperature drying for approximately 1.5 h. Figure 1 (c) shows the current-voltage (I-V) and light output intensity-current (L-I) curves of the InGaN/GaN LEDs with different oxygen plasma-treated times of 0, 1, 5, and 10 s. The InGaN/GaN LEDs with an oxygen plasma-treated time of 5 s represent a similar forward voltage and light output intensity at a driving current of 20 mA. As the oxygen plasma-treated time rises to 10 s, a high forward resistance and a low light output intensity can be observed in Fig. 1 (c). The resistivity of ITO will rise due to a strong ion bombardment damage under a high oxygen plasma-treated time. Conversely, a hydrophilic surface cannot obtain for the oxygen plasma-treated time below 5 s. To reduce the complexity of the experimental process and to obtain the optimal PS NS arrangement for the InGaN/GaN LEDs, optical intensities for the InGaN/GaN LEDs with PS NS array window layers with various PS NS diameters and periods in the *x* and *y* directions were calculated using the finite-difference time-domain (FDTD) method.

### Fabrication of InGaN/GaN blue LEDs with a Periodic PS NS Array on an ITO Layer

The epi-wafers of InGaN/GaN blue LEDs were grown on a c-face (0001) sapphire substrate by using a metal-organic chemical vapor deposition system. The device structure consists of a GaN buffer layer grown at a low temperature, a highly Si-doped n-type GaN layer, an InGaN/GaN multiple quantum wells (MQWs) active region, and an Mg-doped p-type GaN layer. The ITO was deposited on the p-type GaN layer as a transparent conductive layer to spread the injection current. The wafer was then patterned using the standard photolithographic process to define square mesas as the emitting regions by partially etching the exposed ITO/p-GaN/InGaN/GaN MQWs/n-GaN. A Ti/Pt/Au alloy was used as the ohmic contact metal on the p- and n-GaN contact regions, and the wafer was then alloyed in an N_2_ atmosphere for 5 min at 450 °C. The size of the emission window for the InGaN/GaN LEDs with ITO was 300 × 300 μm^2^. The finished wafer was placed in the PS NSs suspension to deposit the compact PS NS array on ITO layer.

## Results and Discussion

**Fig. 2:**
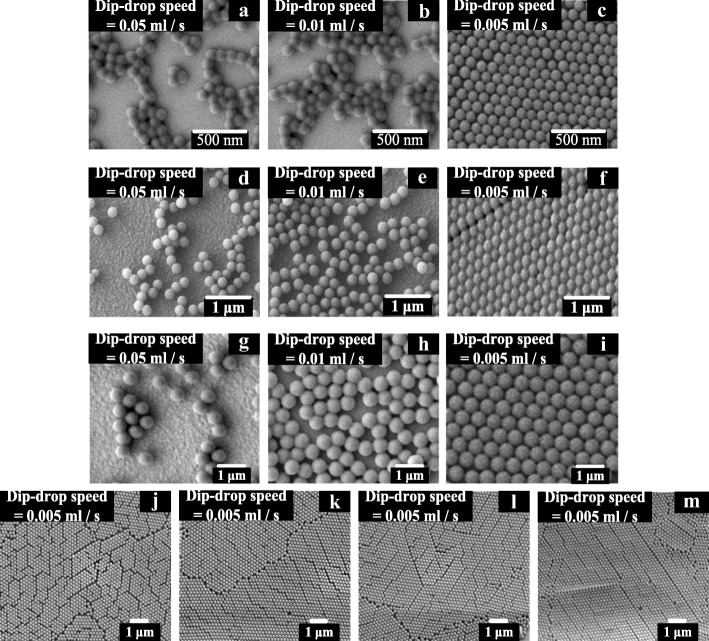
SEM images of PS NSs with diameter of 100, 200, and 500 nm at the average dip-drop speed of **a**, **d**, **g** 0.05 mL/s; **b**, **e**, **h** 0.01 mL/s; and **c**, **f**, **i** 0.005 mL/s, and the SEM images of PS NSs with 200 nm and average dip-drop speeds of 0.005 mL/s at the regions of **j** upper-right, **k** upper-left, **l** lower-right, and **m** lower-left of 0.5 × 0.5 mm^2^ ITO-coated glass substrate . The concentrations of DI water-diluted PS NS suspension were 4.1 × 10^11^ spheres/cm^−3^ for the 100-nm PS NSs, 5.1 × 10^10^ spheres/cm^−3^ for the 200-nm PS NSs, and 3.2 × 10^9^ spheres/cm^−3^ for the 500-nm PS NSs

Fig. 2a–i show scanning electron microscopy (SEM) images of the PS NSs with diameters of 100, 200, and 500 nm, on the ITO coated glass substrate, with average dip-drop speeds of 0.05, 0.01, and 0.005 mL/s. The concentrations of the PS NS suspensions were 4.1 × 10^11^ spheres/cm^−3^ for the 100-nm PS NSs, 5.1 × 10^10^ spheres/cm^−3^ for the 200-nm PS NS, and 3.2 × 10^9^ spheres/cm^−3^ for the 500-nm PS NS. The PS NSs exhibited a widely dispersed distribution on ITO-coated glass substrate under a high average dip-drop speed, but they formed a compact array as the average dip-drop speed was decreased, as shown in Fig. 2. The arrangement of the PS NSs depends on the shape of the liquid surface, which is related to the lateral capillary force [30]. The lateral capillary force can be classified as a floating force or an immersion force. Floating force is caused by the particle weight and Archimedes force, whereas immersion force results from capillary action [31]. During the dip-drop process, the floating force dominated because of the effect of gravity. The floating force can be attractive or repulsive between two PS NSs depending on the shape of the surface between the air and aqueous solution. High average dip-drop speed causes a dramatic perturbation in PS NS suspension near the tuning control valve, and the perturbation results in a convex surface between the air and aqueous solution, leading to a repulsive floating force between two PS NSs. The PS NSs were separated by the repulsive floating force during the dip-drop process, resulting in a disordered PS NS arrangement on the ITO-coated glass substrate, as observed in Fig. 2a, d, g. When the average dip-drop speed was decreased to 0.01 mL/s, the perturbation near the tuning control valve was alleviated, as shown in Fig. 2b, e, h. This weak perturbation caused a low repulsive floating force and yielded a smaller space between two PS NSs than that at the dip-drop speed of 0.05 mL/s. As the average dip-drop speed was decreased to 0.005 mL/s, the shape of the surface between the air and the aqueous solution became concave, generating an attractive floating force between the two PS NSs during the dip-drop process. The attractive floating force can result in a compact PS NS array on the ITO-coated glass substrate, as shown in Fig. 2c, f, i. In addition, PS NSs with diameters of 200 and 500 nm exhibited a more compact arrangement on the ITO-coated glass substrate compared with 100-nm-diameter PS NSs under a similar average dip-drop speed because a concave shape surface between the air and aqueous solution was easily formed for PS NSs with large diameters. When the average dip-drop speed was further reduced to < 0.005 mL/s, the PS NS array fabricated using the dip-drop method became impractical for LEDs because of the low throughput. To find the distribution of the compact PS NSs array on the 0.5 × 0.5-mm^2^ ITO-coated glass substrate, Fig. 2j–m shows the SEM images of 200-nm-diameter PS NSs under the average dip-drop speeds of 0.005 mL/s at the regions of upper-right, upper-left, lower-right, and lower-left of ITO-coated glass substrate. These images represent a uniform distributing and compact PS NSs array over the ITO-coated glass substrate, suggesting that InGaN/GaN LED with a uniform and compact PS NSs array window layer can be proposed by using dip-drop method.

**Fig. 3:**
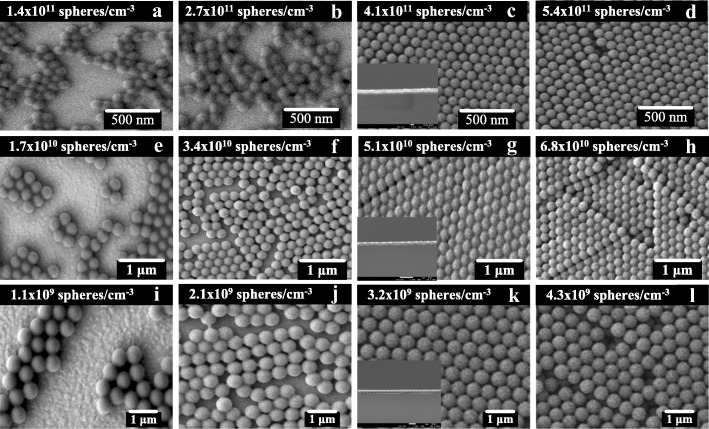
SEM images of PS NSs with PS NS suspension concentrations of **a** 1.4 × 10^11^, **b** 2.7 × 10^11^, **c** 4.1 × 10^11^, and **d** 5.4 × 10^11^ sphere/cm^−3^ for 100-nm PS NSs; **e** 1.7 × 10^10^, **f** 3.4 × 10^10^, **g** 5.1 × 10^10^, and **h** 6.8 × 10^10^ sphere/cm^−3^ for 200-nm PS NSs; and **i** 1.1 × 10^9^, **j** 2.1 × 10^9^, **k** 3.2 × 10^9^, and **l** 4.3 × 10^9^ sphere/cm^−3^ for 500-nm PS NSs at an average dip-drop speed of 0.005 mL/s. The insets of **c**, **g,** and **k** represent the cross-section SEM images of PS NSs with PS NS suspension concentrations of 4.1 × 10^11^ sphere/cm^−3^ for 100-nm PS NSs, 5.1 × 10^10^ sphere/cm^−3^ for 200-nm PS NSs, and 3.2 × 10^9^ sphere/cm^−3^ for 500-nm PS NSs

The concentration of the PS NS suspension also affects the PS NS arrangement and layer number of the PS NS array. PS NS suspensions with high concentrations result in compact PS NS arrays with monolayer or multilayers, whereas suspensions with low concentration might generate loose or compact PS NS arrays with monolayers. Multilayer PS NS arrays have shortcomings such as low transmittance, difficult definition for the PS NS period, and low reliability, making them unsuitable for LED applications. The optimal concentration of the PS NS suspension must be determined to obtain compact monolayer PS NS array. In this study, the concentration of a PS NS suspension was defined as the ratio of the number of PS NSs and the volume of the suspension. Figure 3 shows the SEM images of PS NSs on the ITO-coated glass substrate for various PS NS suspension concentrations: (a) 1.4 × 10^11^, (b) 2.7 × 10^11^, (c) 4.1 × 10^11^, and (d) 5.4 × 10^11^ sphere/cm^−3^ for 100-nm PS NSs; (e) 1.7 × 10^10^, (f) 3.4 × 10^10^, (g) 5.1 × 10^10^, and (h) 6.8 × 10^10^ sphere/cm^−3^ for 200-nm PS NSs; and (i) 1.1 × 10^9^, (j) 2.1 × 10^9^, (k) 3.2 × 10^9^, and (l) 4.3 × 10^9^ sphere/cm^−3^ for 500-nm PS NSs with an average dip-drop speed of 0.005 mL/s. When the concentration of the PS NS suspension was < 4.1 × 10^11^ sphere/cm^−3^ for 100-nm PS NSs, < 5.1 × 10^10^ sphere/cm^−3^ for 200-nm PS NSs, and < 3.2 × 10^9^ sphere/cm^−3^ for 500-nm PS NSs, some areas of the ITO-coated glass substrate were free of PS NSs, as shown in Fig. 3a, b, e, f, i, j. When the concentration was increased to 4.1 × 10^11^ sphere/cm^−3^ for 100-nm PS NSs, 5.1 × 10^10^ sphere/cm^−3^ for 200-nm PS NSs, and 3.2 × 10^9^ sphere/cm^−3^ for 500-nm PS NSs, a compact PS NS array of monolayers covered the ITO-coated glass substrate, as shown in Fig. 3c, g, k. The insets of Fig. 3c, g, k show the cross-section SEM images of PS NSs on the ITO-coated glass substrate under the PS NS suspension concentrations of 4.1 × 10^11^ sphere/cm^−3^ for 100-nm PS NSs, 5.1 × 10^10^ sphere/cm^−3^ for 200-nm PS NSs, and 3.2 × 10^9^ sphere/cm^−3^ for 500-nm PS NSs. A compact PS NS monolayer can be formed on the ITO-coated glass substrate under above concentrations of PS NS suspensions and dip-drop speed. The PS NSs in the high-concentration PS NS suspension were denser than those in the low-concentration PS NS suspension. During the dip-drop process, the attractive floating force formed a compact PS NS array of monolayers and a dispersed PS NS array on the ITO-coated glass substrate under the high- and low-concentration PS NS suspensions, respectively, because insufficient PS NSs were available to cover the ITO-coated glass substrate under the low-concentration PS NS suspension. When the concentration of the PS NS suspension was further increased to 5.4 × 10^11^ sphere/cm^−3^ for 100-nm PS NSs, 6.8 × 10^10^ sphere/cm^−3^ for 200-nm PS NSs, and 4.3 × 10^9^ sphere/cm^−3^ for 500-nm PS NSs, the ITO-coated glass substrate was covered by a compact PS NS array of multilayers because excessively, many PS NSs participated in the deposition. The excess PS NSs reached the surface of the compact PS NS array of the monolayer and then stuck to it to form the compact PS NS array of multilayers.

**Fig. 4:**
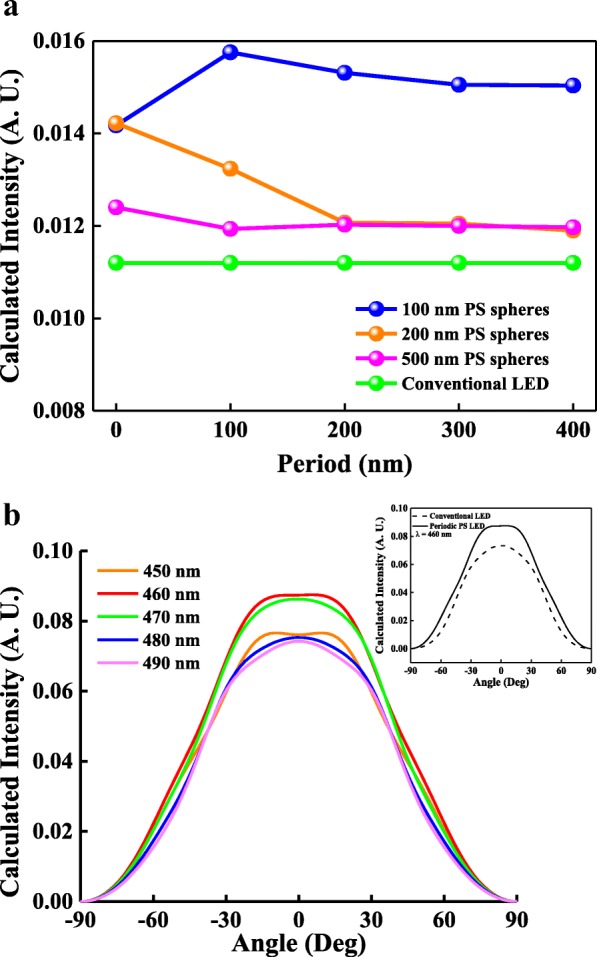
Calculated intensity of **a** conventional LEDs and LEDs with different periods for PS NSs with 100 and 200 nm diameters and (**b**) varied angle under the different emission wavelength. The inset of **b** display the angular spectra of InGaN/GaN blue LED with the optimal periodic PS NS array window layer and without PS NS array window layer under emission wavelength of 460 nm

The light escape cone of an InGaN/GaN LED is limited because of the high refractive index contrast between GaN and air, resulting in a low LEE. Let **k** be the wave vector of the escape cone; then,

1 $$ \mathbf{k}={\mathbf{k}}_{\mathbf{N}}+{\mathbf{k}}_{\mathbf{L}} $$

where **k**_**N**_ and **k**_**L**_ are the wave vectors normal to device and in-plane, respectively. With the periodic PS NS array window layer on InGaN/GaN LED, if the refractive index periodicity of a periodic PS NS array diffracts the wave-guided modes above a certain cutoff frequency into externally propagating modes, the in-plane wave vector changes to **k**_**WG**_ + n**k**_**PS**_, where **k**_**WG**_ is the wave vector of the wave-guided light parallel to the device, and **k**_**PS**_ is the reciprocal wave vector of the periodic PS NS array, given by

2 $$ {\mathbf{k}}_{\mathbf{PS}}=\left(2\pi /{x}_{\lambda}\right){\widehat{\mathbf{a}}}_{\mathbf{x}}+\left(2\pi /{y}_{\lambda}\right){\widehat{\mathbf{a}}}_{\mathbf{y}} $$

where *x*_*λ*_ and *y*_*λ*_ are periods in the *x* and *y* directions of PS NS array. For a periodic PS NS array, the original in-plane wave vector, **k**_**L**_, changes to **k**^**`**^_**L**_ and **k**^**`**^ and can be expressed as

3 $$ {{\mathbf{k}}^{\hbox{'}}}_{\mathbf{L}}={\mathbf{k}}_{\mathbf{L}}+{\mathbf{nk}}_{\mathbf{PS}} $$

where n is an integer. The light escape cone can be improved by changing the periods in the *x* and *y* directions to modulate **k**_**PS**_; thus, the LEE of InGaN/GaN LED can be enhanced by reducing **k**^**´**^_**L**_. However, the optimal periods in the *x* and *y* directions relative to the cutoff frequency to satisfy the emission wavelength of InGaN/GaN blue LED are difficult to obtain through experimental processes. To simplify the investigation, Rsoft software (Cybernet Ltd.), fullwave Sim Add-on Module with three-dimensional FDTD method, and Rsoft LED Utility were used to calculate the extracted light intensity from p-GaN to free space for InGaN/GaN blue LEDs without and with the PS NS array window layers with various periods in the *x* and *y* directions. Figure 4a presents the calculated light intensity as a function of the period for LEDs with PS NS array window layers with 100-, 200-, and 500-nm-diameter PS NSs and conventional InGaN/GaN LEDs. The calculated light intensities for the LEDs with the PS NS window layers (blue, yellow, and red curves) were higher than that for the conventional LEDs as shown in Fig. 4a. In addition, the LED with periodic PS NS arrays of the diameter and the periods in *x* and *y* directions of 100, 100, and 100 nm has the highest calculated light intensity and shows an enhanced factor of 1.4 as compared to the LED without PS NS array. This was because the light escape cone for InGaN/GaN LEDs with periodic monolayer PS NS arrays can be improved by adjusting **k**_**PS**_, thereby enhancing the LEE of InGaN/GaN LEDs with periodic PS NS array window layers. To obtain the maximum light intensity for InGaN/GaN LEDs, the optimal diameter and the periods in *x* and *y* directions for the PS NS array were calculated as 100, 100, and 100 nm. Furthermore, to comprehend the enhanced LEE of InGaN/GaN LEDs with optimal periodic PS NS array related to the diffraction mode, the extracted light intensity from p-GaN to free space for InGaN/GaN blue LED without and with the optimal PS NS array window layers under different emission wavelength and angle was calculated. Figure 4b shows the calculated light intensity as a function of varied angle under the different emission wavelength, and the inset of Fig. 4b displays the angular spectra of InGaN/GaN blue LED with the optimal periodic PS NS array window layer and without PS NS array window layer under emission wavelength of 460 nm. The InGaN/GaN LED with optimal periodic PS NS array emitted at wavelength of 460 nm performs the highest and widest spectrum as compared to those with optimal periodic PS NS array emitted at 450, 470, 480, and 490 nm and InGaN/GaN LED without PS NS array because it satisfies the guide mode diffracted into air by the optimal periodic PS NS array.

**Fig. 5:**
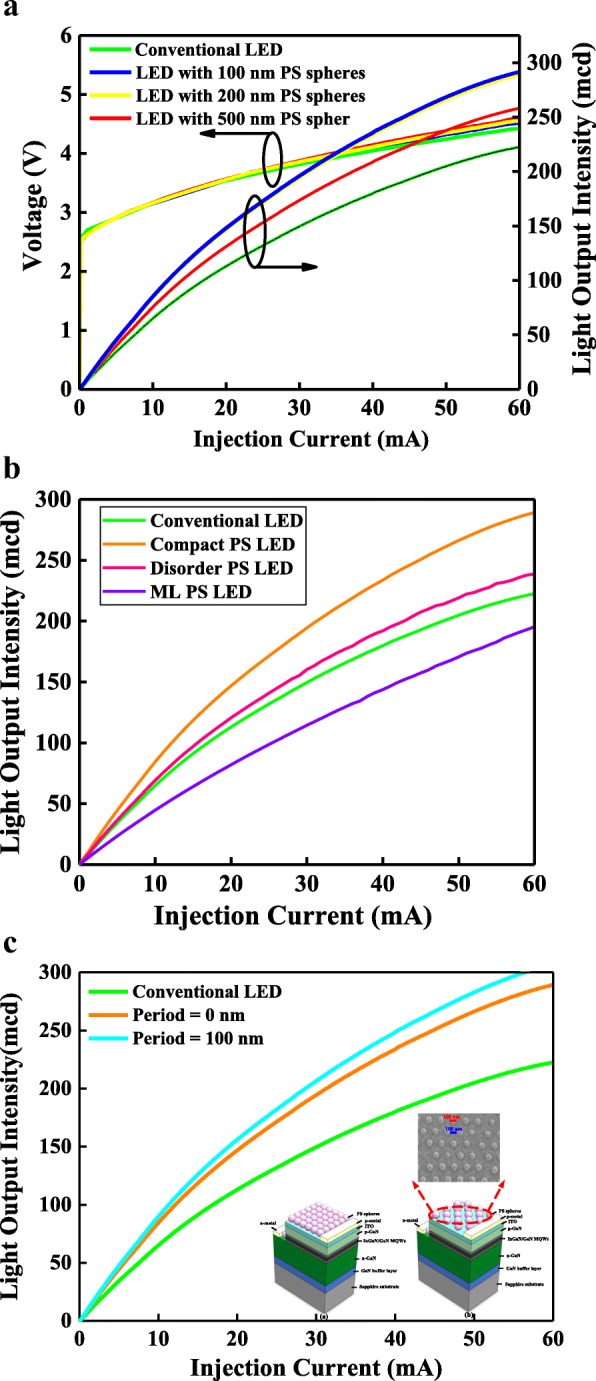
**a** I-V and L-I curves for the conventional InGaN/GaN LED and InGaN/GaN LED with compact PS NS array window layers of 100-, 200-, and 500-nm-diameter PS NSs. **b** L-I curves for the conventional InGaN/GaN LEDs and InGaN/GaN LEDs with periodic, disorder, and multilayer PS array window layers. **c** L-I curves for the conventional InGaN/GaN LEDs and InGaN/GaN LEDs with compact and optimal periodic PS NS array window layers. The inset of (**c**) shows the schematic structures of InGaN/GaN LEDs with compact and periodic PS array. The SEM image of the periodic PS array the also represents in the inset of Fig. 5

Figure 5a shows the I-V and L-I curves of the InGaN/GaN LEDs without and with the window layer formed by a compact monolayer PS NS array of 100-, 200-, and 500-nm-diameter PS NSs. Under the injection current of 20 mA, the forward voltages for InGaN/GaN LEDs without and with the compact PS NS array were 3.54, 3.55, 3.55, and 3.55 V. The similar forward voltages for InGaN/GaN LEDs with and without PS NS array window layers were attributed to them having the same epitaxial structure. Additionally, the forward resistance for InGaN/GaN LEDs without PS NS array window layers was slightly lower than those with PS NS array window layers, because the ITO transparent conduction layer was degraded by the oxygen plasma during the hydrophilic process. The light output intensities for InGaN/GaN LEDs without and with the 100-, 200-, and 500-nm PS NS array window layers were 112.9, 146.8, 148.0, and 131.1 mcd, respectively, as shown in Fig. 5a. The light output intensities of InGaN/GaN LEDs without and with the PS NS array window layer showed trends similar to the calculated results in Fig. 4. The photons emitted from the InGaN/GaN active region underwent TIR at the ITO/air interface because they were outside the light escape cone. However, the InGaN/GaN LEDs with PS NS array window layers changed the in-plane vector (**k**_**L**_^**`**^), resulting in an enhanced LEE; therefore, the light output intensity of InGaN/GaN LEDs with PS NS array window layers can be increased. In addition, the incident angle of the emission light at the interface between the PS NS array and air was affected by the PS NSs because of the nonplanar interface as well as the textural structure. Consequently, the periodic PS NS array window layer enhanced the LEE of the InGaN/GaN LEDs. Figure 5b shows the L-I curves of the conventional InGaN/GaN LED and those InGaN/GaN LEDs with compact, disorder, and multilayer PS array window layers. The light output intensity of InGaN/GaN LED with disorder PS layer shows a slightly higher than conventional InGaN/GaN LED because the photons can be partially out-coupled at the interface of air/ITO by the disorder PS window layer. In addition, the light output intensity of InGaN/GaN LED with multilayer PS array window layer is lower than conventional InGaN/GaN LED because of the low transmittance (< 80%) for the multilayer PS array. Figure 5c presents the L-I curves of the conventional InGaN/GaN LEDs and those with compact and periodic PS NS array window layers. The diameter and the periods in *x* and *y* directions for periodic PS NS arrays were 100, 100, and 100 nm, respectively, satisfying the optimality condition calculated from Fig. 4. The periodic PS NS array can be obtained by etching the compact PS NS array of 200-nm PS NSs, and the inset of Fig. 5c shows the schematic structures of InGaN/GaN LEDs with compact and periodic PS array and SEM image of etched 100-nm PS NS array with periods of 100 and 100 nm in the *x* and *y* directions. The InGaN/GaN LED with the window layer of periodic 100-nm PS NS array with periods in the *x* and *y* directions of 100 and 100 nm exhibited the highest light output intensity, as shown in Fig. 5c, which was in agreement with the calculated results in Fig. 4. The InGaN/GaN LEDs with the optimal periodic PS NS array window layers yielded a 38% increase in light output intensity compared with those without PS NS arrays because of the improved LEE. In addition, insets of Fig. 5c and Fig. 2f indicate that the PS NSs show a well adherent on ITO and a less etching damage during the post-deposition etching process.

Table 1 lists the average forward voltages and light output intensities at the injection current of 20 mA for the selected chips from different position of InGaN/GaN wafers with optimal PS NS array window layers made of three different runs under the same condition. A uniform and reliable arrangement of PS NSs on the InGaN/GaN wafers was extremely noteworthy because this is the main factor affecting the performance of InGaN/GaN LEDs. The period and size of the PS NSs on the InGaN/GaN wafers were relatively similar; the device-to-device standard deviation of measured enhancement of emission intensity was about 1.4%, and the variations were approximately 1.9% for the forward voltage and 2.9% for the light output intensity under the same driving current.

**Table 1:** The average turn-on voltage and light output intensity for selected chips from different position of InGaN/GaN LED wafers with the optimal PS NS arrangement (PS NS diameter of 100 nm and period of 100 nm) on an ITO window layer under a 20-mA driving current

Chip no.	Turn-on voltage (V) at 20 mA	Light output intensity (mcd) at 20 mA
1	3.65	155.9
2	3.65	154.3
3	3.66	153.1
4	3.65	151.8
5	3.72	156.4
6	3.71	154.2
7	3.67	152.4
8	3.68	154.7
9	3.67	153.3
10	3.68	156.1

**Fig. 6:**
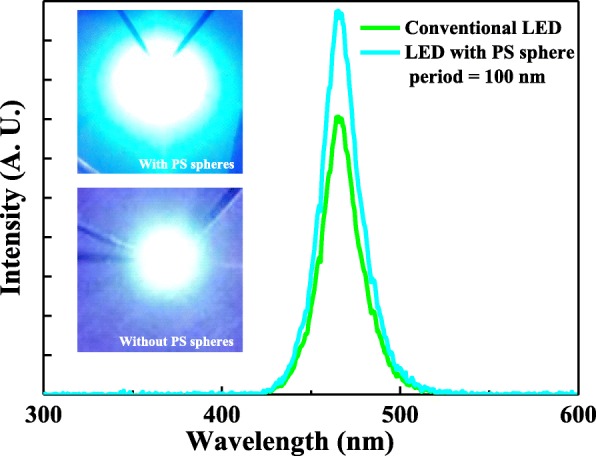
Electroluminescence spectra as functions of wavelength for the conventional InGaN/GaN LEDs and InGaN/GaN LEDs with optimal periodic PS NS array window layers

Figure 6 shows the electroluminescence spectra as a function of wavelength for the conventional InGaN/GaN LEDs and the InGaN/GaN LEDs with the optimal periodic PS NS array window layers under the driving current of 20 mA. The light output intensity at 465.5 nm and full width at half maximum of the emission spectrum for the InGaN/GaN LEDs with the optimal periodic PS NS array window layers were stronger and narrower than those of the conventional InGaN/GaN LEDs. The guided light that is emitted from the InGaN/GaN active region underwent TIR and could not phase match to the radiation modes when the amplitude of the in-plane wave vector in the semiconductor was higher than that in the air [9, 32]. The periodic PS NS array window layers could modulate the amplitude of the in-plane wave vector in the semiconductor to less than that in air, and therefore, the light was emitted from the semiconductor with the periodic PS NS array because the phase of the guided modes matched the radiation modes, resulting in a high light output intensity and a narrow emission spectrum. The insets of Fig. 6 show the micrographs of light emission for the conventional InGaN/GaN LEDs and the InGaN/GaN LEDs with the optimal periodic PS NS array window layers. The light output intensity for the InGaN/GaN LEDs with the optimal periodic PS NS array window layers was higher than that of the conventional InGaN/GaN LEDs because of the improved LEE.

## Conclusion

PS NS array window layers can improve the LEE of InGaN/GaN LEDs. A compact monolayer PS NS array was obtained by adjusting the average dip-drop speed and PS suspension concentration. The optimal average dip-drop speed and PS NS suspension concentration to obtain a compact monolayer PS NS array were 0.005 mL/s and 4.1 × 10^11^ sphere/cm^−3^, respectively, for 100-nm PS NSs; 0.005 mL/s and 5.1 × 10^10^ sphere/cm^−3^, respectively, for 200-nm PS NSs; and 0.005 mL/s and 3.2 × 10^9^ sphere/cm^−3^, respectively, for 500-nm PS NSs. The calculated and experimental results indicated that the periodic PS NS array window layer with PS NS diameter of 100 nm and periods of 100 nm in the *x* and *y* directions effectively enhanced the LEE of the InGaN/GaN LEDs. The InGaN/GaN LEDs with the optimal periodic PS NS array window layer yielded a 38% increase in light output intensity compared with that of the conventional InGaN/GaN LED under a 20-mA driving current because of the high LEE.

## Abbreviations

EQE: External quantum efficiency
FDTD: Finite-difference time-domain
ITO: Indium-tin-oxide
I-V: current-voltage
LED: Light-emitting diode
LEE: Light extraction efficiency
L-I: Light output intensity-current
PCs: Photonic crystals
PS NS: Polystyrene nanosphere
SEM: Scanning electron microscopy
TIR: Total internal reflection
